# Towards biome-specific analysis of meta-omics data

**DOI:** 10.1038/ismej.2015.188

**Published:** 2015-12-01

**Authors:** Youssef Darzi, Gwen Falony, Sara Vieira-Silva, Jeroen Raes

**Affiliations:** 1 Microbiology Unit, Faculty of Sciences and Bioengineering Sciences, Vrije Universiteit Brussel, Brussels, Belgium; 2 VIB, Center for the Biology of Disease, Leuven, Belgium; 3 Department of Microbiology and Immunology, Rega Institute, KU Leuven, Leuven, Belgium

Microbial ecology has witnessed tremendous progress over the last decade empowered by meta-omics approaches and innovations in DNA/RNA sequencing as well as high-resolution mass spectrometry. In this climate, the rise of meta-omics projects (Raes, 2011) such as MetaHIT and the Human Microbiome Project, Tara Oceans, the Global Ocean Sampling Expedition and the Earth Microbiome Project aiming at unraveling the structure and function of specific microbiomes in different habitats was observed. Now that massive data generation is no longer science fiction, the bottleneck shifts to computational analysis (Falony *et al.*, 2015).

On the bioinformatics front, important efforts have already gone into the ‘upstream' part of the analysis. Traditional sequence mapping, assembly, binning and clustering approaches have been scaled up for the handling of hundreds of gigabytes of sequencing data (Kim *et al.*, 2013), and the generation of biome-wide gene catalogs greatly facilitates the analysis. Also, machine learning techniques have been successfully applied on several occasions to predict disease biomarkers from meta-omics data (for example, Williams *et al.* (2009) and Zeller *et al.* (2014)). However, for metabolic pathway-based functional analysis, researchers usually still rely on ‘classic' approaches developed for single genomes. To identify and quantify the biochemical functions and pathways that make up the metabolic wiring of an ecosystem and assess functional shifts upon perturbation, associations between environment, metabolism and species–function relationships, current studies usually rely on broad metabolic databases (for example, KEGG (Kanehisa *et al.*, 2014)). Despite their unquestionable merit, such resources unfortunately tend to be biased, for historical reasons, toward Eukaryotes and model organisms' metabolism. These databases thus often include pathways and pathway variants that do not exist in many ecosystems under research, or lack part of its enzymatic routes, which can be misleading when drawing conclusions and penalizing for statistical significance in large-scale studies. As a case in point, no specific pathway module for the production of butyrate can be found in the KEGG encyclopedia, despite its significant clinical importance for the gut ecosystem. Thus, using ‘universal' databases often results in suboptimal functional assignment and fewer or false-positive outcomes.

Recently, novel approaches are being pursued to improve the sensitivity and specificity of functional interpretation of meta-omics data using biome-specific approaches. For instance, Le Chatelier *et al.* (2013) investigated specific metabolic shifts in the gut metagenome of obese individuals based upon inspection of 51 manually compiled gut-specific pathway modules and Sunagawa *et al.* (2015) studied ocean biochemical processes using a targeted set of markers for essential ocean biogeochemical processes. Likewise, Prestat *et al.* (2014) used manually curated HMM profiles targeted for soil biochemical pathways to improve the accuracy and the rate of functional annotation of soil metagenomic samples. However, such biome-specific approaches are more exceptions rather than the rule.

Here, we illustrate the advantages of using biome-specific approaches in an example comparative analysis of a human gut metaproteomics data set (10 samples: 4 are healthy individuals and 6 Crohn's Disease (CD) patients in remission) from Erickson *et al.* (2012) by comparing the outcome of a standard (KEGG-based) analysis versus GOmixer (Raes Lab, Ghent, Belgium), a human gut-specific metabolic pathway analysis tool that we developed for this purpose (available as an online tool and downloadable software package at: http://www.raeslab.org/gomixer/).

In short, the GOmixer workflow starts by quantifying human gut metabolic pathway modules for each sample, by mapping gene abundances on a database of predefined gut-specific modules. A module is a set of tightly related enzymatic functions that represent a cellular process with defined input and output metabolites. The modules used in GOmixer's database were manually compiled based on extensive literature searches (Le Chatelier *et al.*, (2013); Vieira-Silva *et al.*, unpublished). For a module to be considered present by GOmixer, its coverage (percentage of metabolic steps present) should be higher than a user specified threshold. Module abundance is defined as the average abundance of the metabolic steps covered for this pathway. After quantification, statistically over/under-represented metabolic modules between two groups of samples, in this case Healthy and CD patients are determined using the nonparametric Wilcoxon's rank-sum test, given that metagenomics data are generally distribution free and that the test is robust to outliers. Benjamini–Hochberg's false discovery rate is then used to correct for multiple testing. The results are displayed on a gut-specific global metabolic map to easily highlight trends in functionally related pathways (Figure 1).

Table 1 shows the comparison between both approaches. The results show that agreement between universal and gut-specific analyses can be found on multiple occasions. For instance, both analyses show differential expression of the Glycolysis and the Entner–Doudoroff pathways. However, several modules not relevant to the context of the human gut were also detected as significantly different using universal module-based analysis. For example, the Crassulacean acid metabolism module (M00169), which is a carbon fixation pathway in plants and M00344 (formaldehyde assimilation, xylulose monophosphate pathway), which is specific to yeast but not to prokaryotes, are both found to be down-regulated in CD patients. The reasons for these observations is the existence of enzymes found in more than one metabolic module, causing enzymes of truly present modules to sometimes yield artifact overrepresentation of other modules as well (Ye and Doak, 2009). Besides increasing the false-positive rate, these uninformative modules inflate the number of statistical comparisons to be done and thus penalize true signals when correcting for multiple testing. We explored whether approaches that aim at reducing false-positive rate by finding a minimum number of pathways that can explain all genes observed in a given metagenome can resolve these issues and reveal only gut-relevant pathways. For this reason, we reanalyzed the data using MinPath (Ye and Doak, 2009) and the KEGG database. Although this clearly improved results, non-relevant pathways (for example, the Crassulacean acid metabolism module) were still recovered (Supplementary Table S1). Moreover, when applying this approach on 1267 (human-filtered) metagenomes (healthy, diabetes and CD) used for the construction of the Integrated Gene Catalog (IGC) (meta.genomics.cn/metagene/meta/home) of the human gut microbiome, several non-relevant plant (for example, M00085: Fatty acid biosynthesis, elongation, mitochondria and M00114: Ascorbate biosynthesis, plants, glucose-6P=>ascorbate), human (for example, M00042: Catecholaminebiosynthesis and M00135: GABA biosynthesis, eukaryotes, putrescine=>GABA) and bacterial modules (for example, M00165: Reductive pentose phosphate cycle (Calvin cycle), M00376: 3-Hydroxypropionate bi-cycle) were recruited (see Supplementary Table S2 for details). However, the bigger problem lies not in overpredicting but in missing relevant modules for the collection of a complete overview of the metabolic network at hand. As an example, the GOmixer-based analysis also uncovered specific gut fermentation modules, which highlighted significant downregulation of proteolytic and lipolytic fermentation, polysaccharide degradation, and the production of short-chain fatty acids in CD patients. These metabolic processes are essential for the functioning of the human gut microbiota (Gerritsen *et al.*, 2011) and very relevant for understanding pathomechanisms; however, no detailed module level definition is available for them in the KEGG database.

Overall, this case study illustrates that using universal, generic approaches in microbiome studies are not without risk, and shows that using biome-specific databases provides substantial advantages for ecological and clinical analysis of meta-omics data sets for specialists and non-specialists alike. The reason for this is threefold: First, the specific focus of biome-specific modules allows careful hypothesis generation and tangible data analysis for non-specialists. Second, it allows moving beyond coarse-grained functional assignment to fine-grained module level assignment, which is crucial to associate bacterial species to specific metabolic roles. Third, modules with well-defined input and output compounds are also important to integrate multiple types of omics (for example, integrating metabolomics data) and to model the ecosystem responses to perturbation or predict future behavior in longitudinal studies. This in turn will help us understand the fundamentals of the microbial ecology of a wide range of ecosystems, help us design better diagnostics (both in clinical as environmental applications), and better targeted therapies/interventions. Finally, at the time where meta-omic data generation is becoming available to all, but the analysis and hypothesis generation remains complex, biome-specific, user-friendly tools such as GOmixer will contribute to the reduction of this complexity and help drive omics-based microbial ecology forward.

## Figures and Tables

**Figure 1:**
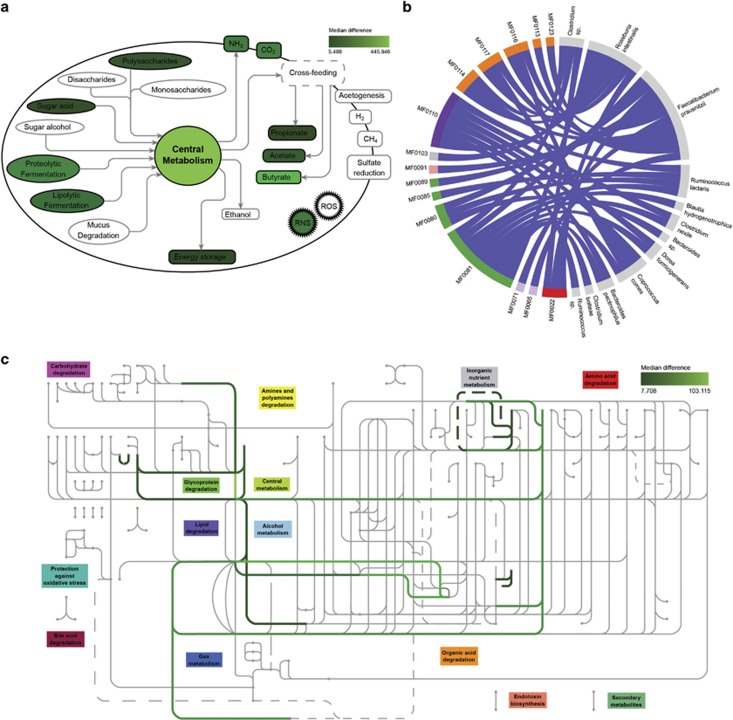
GOmixer analysis outcome of Erickson *et al.* (2012). (**a**) Global gut metabolic processes map that gives an overview of the major metabolic processes in the gut. The color scale reflects significantly enriched abundances in Healthy compared to Crohn's Disease (CD) subjects. (**b**) Chord plot highlighting species-function associations. Modules (MF numbers) belonging to the same global metabolic process share the same color. Association links reflect module over/under-representation in Healthy (blue) or CD (red). In this analysis all functions are over represented in Healthy. (**c**) Gut module map consisting of modules connected by their input and output compounds. They are clustered according to their hierarchical classification (for example, amino-acid degradation) and reflect the flow of compounds from top to bottom. The color scale reflects significantly enriched abundances in Healthy compared with CD subjects.

**Table 1:**
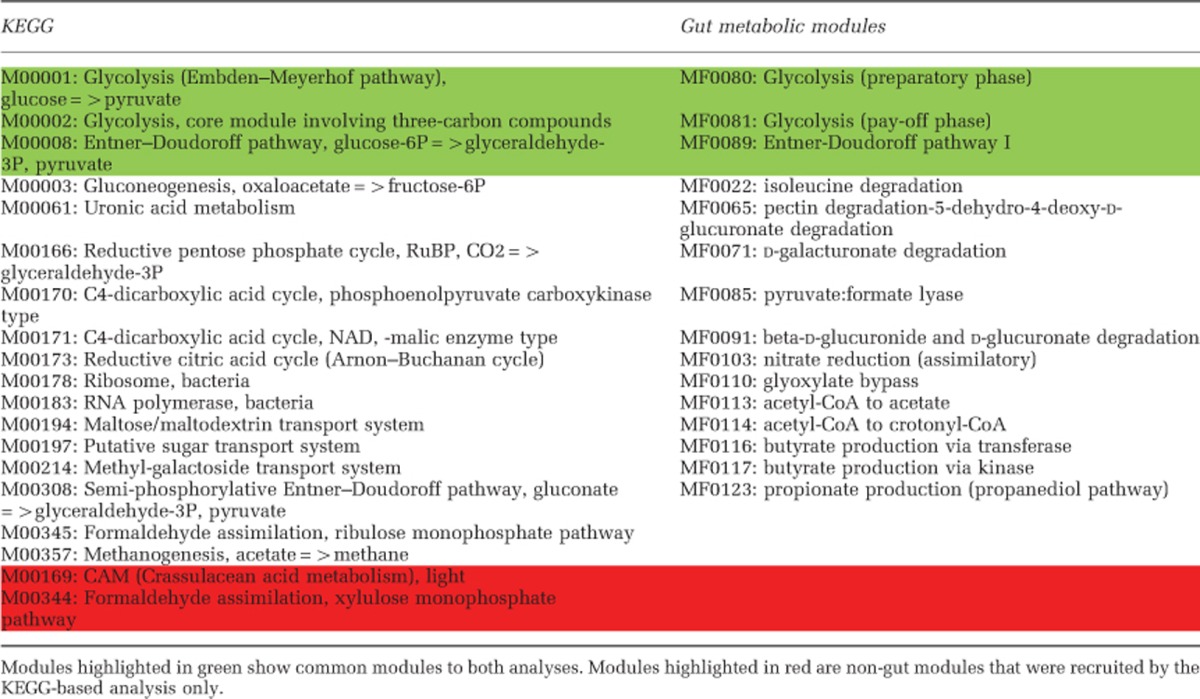
Comparison between KEGG- and GOmixer-based metaproteomics analysis of Crohn's patients vs controls
